# Supplementary material for: Facilitatory rTMS over the Supplementary Motor Cortex Impedes Gait Performance in Parkinson Patients with Freezing of Gait
Source: Brain Sci. 2021 Mar 3;11(3):321. doi: 10.3390/brainsci11030321 (PMC7999694; doi:10.3390/brainsci11030321)
Supplement: Supplementary file 1 [file brainsci-11-00321-s001.pdf]

Supplementary materials

S1 Definition of the OFF and ON state

The *OFF state* was defined as the time after >8 hours overnight drug withdrawal. Dopamine agonists were interrupted for >1 day, MAO-B-inhibitors treatment for >2 days. In PD patients under levodopa monotherapy (+/- COMT inhibitors) with clear and expectable wearing-offs, we alternatively offered to record their hourly motor performance over three subsequent days in a diary. When a clear pattern of consistent wearing-offs was observed, we scheduled their appointments according to the expected OFF time. Thus we were able to reduce the patients' discomfort to travel to our centre in the OFF. Upon arrival, the OFF was evaluated by repeatedly performing representative tasks of the UPDRS (bradykinesia, tremor, rigor, walking). When the patients motor performance did not deteriorate further over >30 minutes, we assumed that the patient had reached the OFF. This approach was only considered in patients with longer journeys and was eventually applied in one patient. All other visits were scheduled early in the morning after overnight drug withdrawal.

The *ON state* was defined as the time 1 hours after the intake of their usual dopaminergic drugs. Again, treatment response was evaluated by repetitively performing indicative tasks of the UPDRS (see above).

Table – S1: gait performance before and after stimulation (sham, iTBS)

gait parameter		before sham	after sham	before iTBS	after iTBS
**P-I (walking without obstacles)**					
stride time (sec)	more affected side	1.01 +/- 0.06	1.01 +/- 0.07	1.01 +/- 0.07	1.01 +/- 0.06
	less affected side	1.01 +/- 0.06	1.01 +/- 0.07	1.01 +/- 0.07	1.01 +/- 0.07
stride length (m)	more affected side	1.13 +/- 0.18	1.17 +/- 0.16	1.13 +/- 0.19	1.14 +/- 0.20
	less affected side	1.13 +/- 0.18	1.17 +/- 0.16	1.13 +/- 0.18	1.14 +/- 0.20
DLST (%)	more affected side	45.44 +/- 5.41	45.25 +/- 5.15	46.25 +/- 5.55	45.28 +/- 5.85
	less affected side	45.52 +/- 4.81	44.97 +/- 4.95	45.47 +/- 4.89	45.50 +/- 5.58
walking speed (m/sec)	more affected side	1.13 +/- 0.19	1.17 +/- 0.19	1.13 +/- 0.21	1.14 +/- 0.21
	less affected side	1.12 +/- 0.19	1.17 +/- 0.19	1.13 +/- 0.21	1.14 +/- 0.21
gait asymmetry	bilateral	4.25 +/- 3.87	4.58 +/- 4.00	4.96 +/- 6.28	4.90 +/- 3.82
**P-II (passing narrow spaces)**					
stride time (sec)	more affected side	1.08 +/- 0.10	1.02 +/- 0.08	1.05 +/- 0.10	1.03 +/- 0.08
	less affected side	1.07 +/- 0.10	1.02 +/- 0.08	1.06 +/- 0.10	1.03 +/- 0.09
stride length (m)	more affected side	1.07 +/- 0.22	1.10 +/- 0.18	1.06 +/- 0.14	1.07 +/- 0.16
	less affected side	1.06 +/- 0.21	1.11 +/- 0.18	1.07 +/- 0.14	1.08 +/- 0.15
DLST (%)	more affected side	47.69 +/- 7.38	45.50 +/- 6.74	47.00 +/- 5.01	46.47 +/- 5.12
	less affected side	46.86 +/- 6.16	45.69 +/- 6.22	47.39 +/- 4.68	45.42 +/- 4.58
walking speed (m/sec)	more affected side	1.01 +/- 0.24	1.09 +/- 0.21	1.02 +/- 0.18	1.05 +/- 0.17
	less affected side	1.01 +/- 0.23	1.10 +/- 0.22	1.02 +/- 0.18	1.06 +/- 0.17
gait asymmetry	bilateral	5.84 +/- 7.57	4.58 +/- 3.53	5.85 +/- 5.24	5.94 +/- 6.27

gait parameter	side of leg	before sham	after sham	before iTBS	after iTBS
**P-III (turning towards more affected side)**					
stride time (sec)	more affected side	0.95 +/- 0.18	0.94 +/- 0.19	1.02 +/- 0.23	0.95 +/- 0.20
	less affected side	0.96 +/- 0.19	0.97 +/- 0.23	1.03 +/- 0.23	0.95 +/- 0.20
stride length (m)	more affected side	0.35 +/- 0.11	0.33 +/- 0.13	0.40 +/- 0.16	0.35 +/- 0.12
	less affected side	0.37 +/- 0.17	0.36 +/- 0.18	0.38 +/- 0.16	0.35 +/- 0.16
DLST (%)	more affected side	57.92 +/- 8.05	60.33 +/- 9.47	58.22 +/- 7.77	60.53 +/- 7.98
	less affected side	57.39 +/- 9.89	59.92 +/- 10.85	58.50 +/- 5.53	59.97 +/- 8.95
walking speed (m/sec)	more affected side	0.37 +/- 0.10	0.35 +/- 0.12	0.39 +/- 0.12	0.37 +/- 0.12
	less affected side	0.38 +/- 0.14	0.37 +/- 0.16	0.38 +/- 0.15	0.36 +/- 0.15
gait asymmetry	bilateral	14.08 +/- 10.76	20.46 +/- 19.43	17.77 +/- 11.86	15.62 +/- 18.03
**P-IV (turning away from more affected side)**					
stride time (sec)	more affected side	0.98 +/- 0.11	0.96 +/- 0.15	1.06 +/- 0.15	0.99 +/- 0.12
	less affected side	0.99 +/- 0.11	0.96 +/- 0.14	1.04 +/- 0.13	0.97 +/- 0.12
stride length (m)	more affected side	0.40 +/- 0.15	0.38 +/- 0.19	0.40 +/- 0.14	0.39 +/- 0.15
	less affected side	0.40 +/- 0.10	0.38 +/- 0.12	0.38 +/- 0.11	0.38 +/- 0.13
DLST (%)	more affected side	55.14 +/- 8.09	56.89 +/- 7.56	55.17 +/- 5.30	56.33 +/- 6.77
	less affected side	55.64 +/- 9.28	56.31 +/- 8.41	56.19 +/- 8.34	58.64 +/- 7.55
walking speed (m/sec)	more affected side	0.40 +/- 0.14	0.39 +/- 0.18	0.39 +/- 0.14	0.39 +/- 0.14
	less affected side	0.40 +/- 0.10	0.40 +/- 0.11	0.37 +/- 0.11	0.39 +/- 0.12
gait asymmetry	bilateral	14.02 +/- 11.28	12.82 +/- 11.84	17.04 +/- 16.23	17.30 +/- 11.75

Global motor performance					
		**before sham**	**after sham**	**before iTBS**	**after iTBS**
UPDRS III (blinded)		29.17 +/- 7.59	26.00 +/- 5.83	28.50 +/- 8.85	26.08 +/- 7.17
UPDRS III (unblinded)		36.67 +/- 7.79	35.17 +/- 7.93	36.75 +/- 7.91	34.42 +/- 7.62

Legend (table S1): The means values (+/- standard deviation) of the respective outcome parameter are provided for each parcour and for global motor performance (UPDRS). Abbreviations: DLST – relative double leg support time; iTBS – intermittent theta burst stimulation; UPDRS – Unified Parkinson's Disease Rating
